# No evidence of mid-flexion instability after robotic-assisted total knee arthroplasty as assessed by intraoperative pressure sensors

**DOI:** 10.1186/s42836-024-00253-3

**Published:** 2024-07-01

**Authors:** Mateo Armendariz, Baha John Tadros, Dermot Collopy, Gavin Clark

**Affiliations:** 1 St John of God Hospital Subiaco and Midland, Subiaco, WA 6008 Australia; 2 University of Western Australia, 35 Stirling Hwy, Crawley, WA 6009 Australia; 3 Perth Hip and Knee Clinic, 1 Wexford St, Subiaco, WA 6008 Australia

**Keywords:** Total knee arthroplasty, Instability, Mid-flexion, Robotic-assisted, Sensors

## Abstract

**Purpose:**

Mid-flexion instability has been identified as a cause for dissatisfaction following total knee arthroplasty (TKA). Robotic-assisted surgery using the Mako robot only allows for assessment of stability at 10° and 90°. This study aimed to investigate any evidence of mid-flexion instability in Mako-assisted TKA.

**Methods:**

Data from 72 TKA in 59 patients from 2018 to 2022 were collected. All patients underwent an RA (Mako, Stryker, Fort Lauderdale, FL, USA), single-radius design, cruciate-retaining TKA. Intraoperatively, medial, and lateral pressures were measured at 10°, 45° and 90° of flexion using a pressure sensor (Verasense, OrthoSensor, 59 Inc., Dania Beach, FL, USA). The knee was considered balanced if the difference in pressures between compartments was less than 15 pounds-force (lbf).

**Results:**

There was no significant difference between the pressures measured in the medial compartment at 10°, 45° and 90° of flexion (*P* = 0.696). A statistically significant difference was found between the pressures measured in the lateral compartment at 10°, 45° and 90° of flexion, with the 10° value being significantly higher (*P* < 0.001), but this did not exceed the threshold of 15 lbf. None of the patients had a pressure difference of more than 15 lbf when pressures at 45° were compared to that at 10° and 90°, medially or laterally.

**Conclusion:**

This study showed no evidence of mid-flexion instability in Mako-assisted TKA, using a single radius, cruciate-retaining prosthesis whilst maintaining the joint height.

**Level of evidence:**

Level III retrospective cohort study.

## Introduction

Achieving stability is paramount to a successful total knee arthroplasty (TKA). Despite technological and technical advances in TKA, instability remains one of the major indications for revision and accounts for 5.6% to 9.6% according to major joint registries [1, 2].

Extension and flexion instabilities have previously been defined and their treatment has been thoroughly discussed in the literature [3]. Mid-flexion instability (MFI) was first described in the early 90’s as a distinct clinical entity in TKA [4]. The most accepted definition of MFI is a clinically unstable knee in the mid-range of motion (10°–90°) [5]. Causes for MFI include component design (multi-radii or posterior-stabilized), surgical mal-positioning and raising the joint line. Patients usually present with feelings of instability, recurrent effusions, difficulty with stair descent or ascent, and soft tissue pain due to hamstring overload. Despite this, the presence of MFI as a separate entity remains disputed given the lack of an objective measure to test for it [4, 6–9].

The introduction of navigated and robotic-assisted (RA) TKA has allowed us to assess for gaps and imbalance intraoperatively and in real-time throughout the range of motion including the mid-range (10°–90°). Examples of this include: VELYS™ Robotic-Assisted Solution (DePuy Synthes, Warsaw, IN, USA), the Brainlab system (Brainlab AG, Heimstetten, Germany), and OrthoMap Precision Knee Navigation (Stryker, Mahwah, NJ, USA). The Mako robot (Stryker, Fort Lauderdale, FL, USA) on the other hand, only allows for the assessment of flexion and extension gaps at 10° and 90°, with no specific assessment of stability during the mid-flexion range. This has raised concerns regarding the risk of mid-flexion instability in patients undergoing TKA using the Mako robot. The literature is lacking in studies confirming or disproving the presence of MFI in Mako-assisted TKA. We aimed to look for any evidence of MFI when a Mako-assisted, single radius design, cruciate-retaining TKA system (Triathlon, Stryker, Mahwah, NJ, USA) is used whilst maintaining the joint height. Several methods were reported in the literature that help quantify gaps and stability intraoperatively [5]. In this study, we used a pressure sensor (Verasense, OrthoSensor, 59 Inc., Dania Beach, FL, USA), which is a disposable tibial insert that provides pressure reading and information regarding implant position in real-time. Several studies have successfully used pressure sensors to assess mid-range instability intraoperatively [10, 11], but this has not been done with the Mako robot before. This was the first study to assess MFI in Mako-assisted TKA using pressure sensors intraoperatively, and we hypothesized that no significant difference would be found in the mid-flexion range pressures compared to flexion or extension.

## Materials and methods

### Patients

The data were retrospectively reviewed of patients undergoing an RA TKA for primary osteoarthritis between May 2018 and May 2022. All procedures were performed by a single surgeon (G.C.) at St John of God Hospital-Perth. Patients were consented and data were collected and stored in a prospective, ethics committee-approved registry.

Seventy-two TKAs were examined in our study. Ten patients received a simultaneous bilateral TKA, and three patients underwent a staged bilateral TKA. The indication for all surgeries was symptomatic primary osteoarthritis. Exclusion criteria included patients with post-traumatic arthritis, posterior-stabilized components, or those having undergone previous surgery (high tibial osteotomy). No subjects were excluded based on age, gender, or body mass index (BMI). Patient demographics are summarized in Table 1.

**Table 1:** Patient-reported outcomes measures

Categories	Outcomes
Total No.	72 TKA
Mean Age (SD)	64 (6.8)
Gender (M/F)	35/37
Mean BMI (SD)	30.7 (5.7)
Alignment (MA/FA)	32/40
Mean Pre-op FJS-12 (SD)	19.3 ± 20.5
Mean Preop OKS (SD)	26.4 ± 7.8

### Surgical technique

Preoperative computer tomography (CT) scans were segmented for interpretation by the Mako software (Mako, Stryker, Fort Lauderdale, FL, USA), which generates a three-dimensional model of the knee, allowing for the virtual placement of the components for preoperative planning. Optical tracking arrays were secured. A medial para-patellar approach was used. Once bone registration was completed, accessible osteophytes were removed, with posterior osteophytes remaining until bone cutting was completed. Soft-tissue laxity was assessed by measuring maximal virtual gaps medially and laterally in extension and flexion with manually stressed poses. The extension poses were taken between 5° and 20° of flexion to remove the tension on the posterior capsule and minimize any effect of posterior osteophytes, and flexion poses were taken at 90°. Cuts were then adjusted, depending on the planned alignment philosophy for the patient (mechanical or functional), and soft tissue releases were performed as needed. In a mechanically-aligned TKA, femoral cuts were made 8 mm from the most distal and posterior points of the condyle with neutral valgus, parallel to the trans-epicondylar axis (TEA) and femoral flexion between 0–7 for best for of the component. The tibial resection of 7 mm from the highest point with neutral coronal alignment, 3 posterior slope and rotation set to Akagi’s line. In a functionally-aligned TKA, a kinematic preliminary plan was employed, whereby equal resections were made in the distal femur medially, and laterally (6.5 mm) and posteriorly (6.5 mm). For the tibial cut, we would aim for 7 mm resections medially and laterally. The posterior slope would be set to be parallel to the lateral tibial plateau slope. Boundaries for functionally-aligned TKA are summarized in Table 2. In all cases, the surgeon targeted 20 mm gaps to allow for a space of 18.5 mm of component depth (with 10 mm insert) and minimal laxity. Femoral resection depths were maintained within 1 mm of original plan to maintain joint line height.

**Table 2:** Functional alignment (FA) boundaries

Categories	Outcomes
Coronal limits	
Hip knee ankle angle	−6° to +3°
Femoral component	+6° to −3°
Tibial component	−6° to +3°
Sagittal limits	
Femoral flexion	0° to 7°
Tibial slope	0° to 7°
Combined component flexion	10°
Femoral rotation (from surgical epicondylar axis)	−6° to +6°

All patients were implanted with an uncemented, cruciate-retaining (CR), fixed bearing design, single-radius femoral component and resurfacing of the patella (Triathlon Stryker, Mahwah, NJ, USA).

### Sensor usage and balance definition

**Fig. 1:**
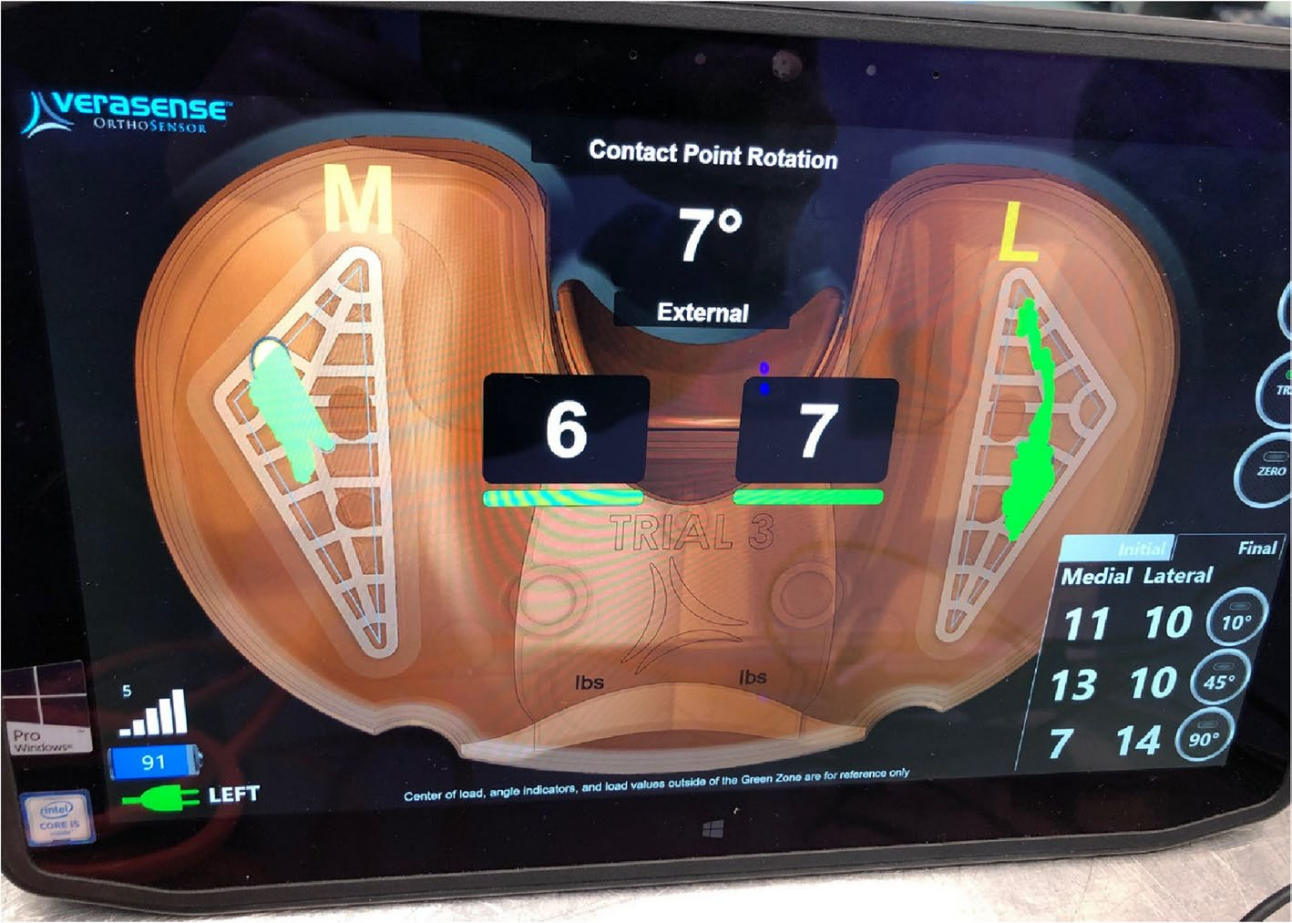
Verasense pressure and kinetic sensor

After insertion of the definitive implants, the pressure sensor was inserted, and the capsule was closed provisionally using clips. The surgeon raised the leg by the heel and the assistant held the thigh, to ensure that no axial load was applied. A knee was considered balanced when it met the criteria outlined by Gustke et al. [12–14]. Intraoperative medial and lateral pressures were obtained at 10°, 45° and 90° of flexion (Fig. 1). The knee was considered “balanced” if the difference in compartment pressures (medial-lateral) was less than 15 pounds-force (lbf).

### Outcomes

Our primary outcome of interest was the pressures recorded intraoperatively using the pressure sensor at 10°, 45° and 90° of flexion. Patient-reported outcomes measures (PROMs) were also collected routinely at the preoperative visit and postoperatively. This included the Forgotten Joint Score 12 (FJS-12) [15] and the Oxford Knee Score (OKS) [16]. Minimum follow-up available for this study was 12 months.

### Statistical analysis

ANOVA analysis of variance was made to compare the pressures measured at different flexion angles. Pressures differences of 15 lbf or more were considered meaningful. The data for demographics and PROMs were presented as means and standard deviations. A *P*-value < 0.05 was considered significant.

## Results

**Fig. 2:**
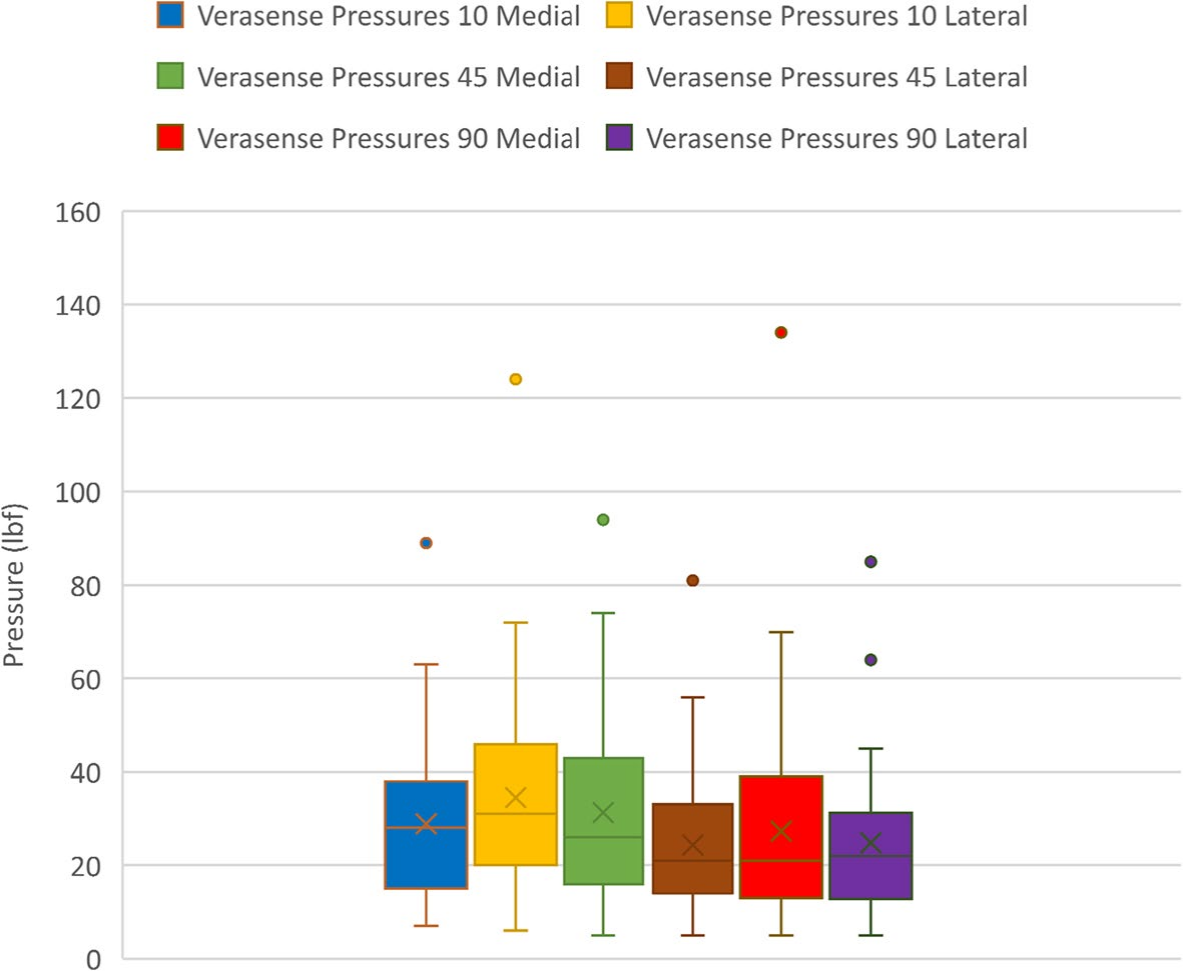
Recorded pressures at 10°, 45°, and 90°

Pressure results for the medial and lateral compartment at 10°, 45° and 90° of flexion are summarized in Table 3. There was no significant difference between the pressures measured in the medial compartment at 10°, 45° and 90° of flexion (*P* = 0.696). The lateral compartment pressures were significantly higher at 10° compared to 45° and 90° (*P* < 0.001). This indicated that the lateral compartment was looser in flexion. The differences in pressure, however, did not exceed the “unbalanced” threshold of 15 lbf (Fig. 2).

**Table 3:** Pressure sensor readings

**Medial compartment flexion**	**Medial (mean** ± **SD)**	**Lateral (mean** ± **SD)**
10°	31.1 ± 20.4	32.6 ± 23.1
45°	30.0 ± 18.9	24.1 ± 16.1
90°	28.6 ± 19.9	22.3 ± 15.5

None of the patients had a pressure difference of more than 15 lbf when pressures at 45° were compared to that at 10° and 90°, medially or laterally, showing no evidence of MFI. The alignment philosophy exerted no impact on the final balancing of the knee (Table 4).

**Table 4:** Mean pressures for MA & FA at 10°, 45°, and 90°

	**MA (SD)**	**FA (SD)**	**Mean difference (95% CI)**	***P*-value**
Medial 10°	35.9 (22.8)	28.9 (17.1)	7.1 (2.5–16.6)	0.143
Lateral 10°	31.3 (23.8)	34.5 (22.3)	3.2 (−7.7–14.2)	0.557
Medial 45°	29.3 (16.9)	31.3 (20.1)	1.9 (−6.9–10.9)	0.661
Lateral 45°	26.2 (15.6)	24.4 (15.7)	1.8 (−9.4–5.8)	0.636
Medial 90°	31.0 (14.6)	27.3 (23.2)	−3.7 (−13.1–5.7)	0.438
Lateral 90°	20.7 (13.4)	24.8 (16.6)	4.1 (−3.1–11.5)	0.259

Patients showed a significant improvement in mean PROMs. FJS-12 had a mean difference of 47.9 (95% CI: 40.9–55), and for OKS the mean difference was 15.9 (95% CI: 13.5–18.4), as shown in Table 5.

**Table 5:** Summary of PROMs

	**Preoperative** **mean (mean** ± **SD)**	**Postoperative 2Y** **mean (mean** ± **SD)**	**Mean difference (95% CI)**	***P*-value**
FJS-12	19.3 ± 20.5	66.0 ± 29.1	47.9 (40.9–55.0)	< 0.001
OKS	26.4 ± 7.8	42.2 ± 6.4	15.9 (13.5–18.4)	< 0.001

## Discussion

Our study showed no evidence of mid-flexion instability at 45°. No significant difference was found in the medial compartment pressures and there was no overload. The lateral compartment was looser at 45° and 90° compared to 10°. This, however, never reached the unbalanced threshold of 15 lbf. The 3D-CT planning allows the surgeon to virtually position the components preoperatively. Intraoperatively, the knee was balanced and stability at 10° and 90° of flexion was achieved. The stability during mid-flexion was not assessed by the Mako software but we evaluated the mid-range balance by measuring compartment pressures at 10°, 45° and 90° of flexion using the Verasense pressure sensor.

Achieving a balanced TKA is crucial to the success of the procedure and improving patient satisfaction [17]. The use of pressure sensors has shown to be a reliable tool for assessing balance in TKA throughout the range of motion. Gustke et al. studied the outcomes of 135 TKAs balanced using the Verasense sensor and showed significant improvement in patient outcomes [12]. Our cohort of patients had a looser lateral compartment in 45° and 90° of flexion relative to the medial side. This closely resembled normal knee kinematics leading to a medial pivot and femoral roll-back. Cochetti et al. used kinetic sensor technology to reproduce such kinematics reliably with good outcomes [10]. This was also employed successfully by Sabatini et al. and, in their study, they recommended using this technology when faced with difficulties in balancing a TKA [18]. In a systematic review comparing Verasense-assisted TKA to manually-balanced TKA, no difference was found in functional outcomes or re-operation rates, but they did show a lower rate of manipulations under anaesthetic (MUA) following sensor-assisted TKA [19].

In all patients in this study, the joint height has been maintained to within 1 mm. Martin and Whiteside first described MFI as a consequence of raising the joint line. In their cadaveric study they showed that moving the joint line 5 mm proximally and 5 mm anteriorly to its anatomical position changed the center of rotation of the femur relative to the collateral ligament attachments and provoked less tension in mid-flexion, with no change of tension in extension and 90° of flexion [4]. Luyckx et al. corroborated these results in a cadaveric study of 10 non-arthritic knees, showing evidence of MFI if the joint line was raised > 2 mm [9]. Konig et al., on the other hand, revealed no evidence of MFI in a computational modeling study when raising the joint line. By their own admission, this conclusion would apply only to the Columbus (Columbus UC, Aesculap AG, Tuttlingen, Germany), posterior-stabilized, ultra-congruent design, which compensated for any laxity in the mid-flexion [20].

We found no difference in pressure when alignment techniques were compared (MA vs. FA) in this cohort of patients. This is similar to the findings of Luyckx et al. when they compared kinematically-implanted TKA to mechanically-aligned ones [9].

The use of a single-radius design has been shown to maintain collateral isometry throughout the range of motion in a study of 10 patients receiving bilateral TKAs, with a single-radius design in one knee and a Multi-radius design in the other [21]. Wang et al., in a kinematic study, showed evidence of medio-lateral instability at the mid-flexion range in multi-radius design knee compared to a single-radius design [22]. Other studies maintained that the design of the prostheses had no bearing on MFI, and, in fact, is related to unrecognized ligamentous laxities and malpositioning (Posterior cruciate ligament and medial collateral ligament) [23]. Our study exhibited no evidence of MFI with the use of a single-radius design. The robot also has the advantage of assessing ligamentous laxity intraoperatively, which could be another explanation for the lack of MFI in our series.

This is, to our knowledge, the first study to assess the evidence of MFI in Mako-assisted TKA. The use of pressure sensors provided an objective tool for our assessment. In addition, the pressures were checked by a single operator using the same technique, which minimized operator discrepancy. There were, however, several limitations to our study. Although the use of pressure sensors is well established in achieving “balance”, the criteria used to define a “balanced knee” were based on retrospective reviews with short-term outcomes [13, 14]. In addition, the balancing aims and targets might differ from other surgeons. The ultimate aim of what constitutes a balanced TKA remains variable amongst surgeons with no consensus reached on the best “end-game”. In this series, the senior author preferred a trapezoidal flexion gap with a lateral side looser to best replicate normal knee kinematics as opposed to a rectangular gap. The study assessed functional outcomes in the short-term with no specific assessment of instability postoperatively. The patients in the study, however, reported excellent FJS-12 scores which does include indirect assessment of instability [15].

## Conclusion

This study showed no evidence of instability in the mid-range as demonstrated by pressure sensors intraoperatively. When performing a Mako-assisted TKA using a single-radius, cruciate-retaining design and maintaining the joint height, checking gaps at the mid-range is unnecessary as the mid-flexion instability is not a concern.

## Data Availability

The datasets used and/or analyzed during the current study are available from the corresponding author on reasonable request.

## Abbreviations

TKA: Total knee arthroplasty
MFI: Mid-flexion instability
RA: Robotic-assisted
CT: Computed tomography
CR: Cruciate retaining
lbf: Pounds-force
PROMs: Patient reported outcomes measures
FJS-12: Forgotten joint score-12
OKS: Oxford knee sore
MA: Mechanical alignment
FA: Functional alignment
